# Prevalence and correlates of burnout among Lebanese health care workers during the COVID-19 pandemic: a national cross-sectional survey

**DOI:** 10.1186/s40545-022-00503-2

**Published:** 2022-12-16

**Authors:** Dalal Youssef, Edmond Abboud, Linda Abou-Abbas, Hamad Hassan, Janet Youssef

**Affiliations:** 1 Bordeaux Research Center for Population Health, Institut de Santé Publique, d’épidémiologie et de Développement (ISPED), Bordeaux University, Bordeaux, France; 2 Clinical Trial Program, Ministry of Public Health, Beirut, Lebanon; 3 Preventive Medicine Department, Ministry of Public Health, Beirut, Lebanon; 4 Ministry of Public Health, Beirut, Lebanon; 5 Neuroscience Research Center, Lebanese University, Faculty of Medical Sciences, Beirut, Lebanon; 6 Al Zahraa Hospital University Medical Center, Beirut, Lebanon

**Keywords:** Burnout, Prevalence, Correlates, COVID-19 pandemic, Health care workers, Cross-sectional

## Abstract

**Background:**

The COVID-19 pandemic has harshly burdened the healthcare systems. Health care workers (HCWs) are at substantial risk of infection and confronted several stressors as well leading them to experience burnout. This study aimed to assess the prevalence of burnout among Lebanese health HCWs and to identify its associated factors.

**Methods:**

A cross-sectional online survey was conducted between the first of November and the end of December 2020 among Lebanese HCWs working in all active hospitals operating across the country. Data were collected using an Arabic, anonymous, self-reported questionnaire comprising four sections: (a) basic sociodemographic characteristics, (b) exposure to COVID-19 covariates, (c) occupational factors, and (d) the measurements including the Copenhagen Burnout Inventory (CBI). CBI subscale cut-off score of 50 was used to assess the prevalence of burnout among HCWs. Multinomial logistic regression analyses were performed to examine the factors associated with the different aspects of burnout.

**Results:**

Out of the 1751 respondents, personal burnout (PB) was detected in its moderate and high-level aspects among 86.3% of Lebanese HCWs. Moderate and high levels of work-related burnout (WB), and client-related burnout (CB) hit 79.2% and 83.3% of HCWs, respectively. HCWs who were females, married, physicians, having a poor health status and specific living conditions (dependent child, elderly at home, family member with comorbidities, and a low income) were more likely to exhibit a high level of PB compared to no/low burnout level. Moreover, frontline HCWs, those infected by COVID-19 or those having a colleague infected by COVID-19, and those exhibiting a high perception of threat were more prone to experience a high-level PB rather than a low/no burnout. Working in a public hospital, extensive working hours, and insufficient sleeping hours were also found positively associated with high PB. However, older age and altruism were negatively associated with high PB compared to no/low PB among HCWs. As for WB, similar factors were found either positively or negatively associated with a high level of WB except health status and living conditions factors (dependent child or family member). As for CB, older age of HCWs (> 30 years) and altruism were negatively associated with high CB compared to no/low burnout level. However, working in the frontline, high threat perception, extensive working hours, insufficient sleeping hours, and low income were positively associated with high CB among HCWs compared to no/low burnout.

**Conclusion:**

The prevalence of burnout among Lebanese HCWs during the pandemic was significant and alarming. Enacting and implementing preventive policies and effective interventions are urgently needed to cultivate wellness among HCWs.

## Background

The ongoing, devastating, and massive global spread of Coronavirus Disease 2019 (COVID-19) has seriously affected all aspects of the daily life of the general population across the globe [1–3]. It has also threatened the people’ mental health. Several studies conducted in the era of COVID-19 recognized the surge in new cases of depression and anxiety and an exacerbation of existing mental health issues among exposed populations, particularly among healthcare workers (HCWs) [4]. A multi-country study assessing the mental health impacts of COVID-19 on HCWs in the Eastern Mediterranean Region (EMR) showed that 57.5% of them had depression, 42.0% had stress, and 59.1% had anxiety [5]. Another study conducted among Jordanian HCWs showed that job stress, staff and resource adequacy, fear of COVID-19 infection, and interprofessional relationships in healthcare practice were key factors to HCWs burnout [6]. Although the documentation of such detrimental impact of the pandemic on mental health, there is a lack of proactive actions in terms of psychological care assistance for those suffering from this crisis [7, 8].

Indeed, burnout emerged as one of the main mental health issues fueled by the pandemic [9]. This syndrome was defined as emotional and physical exhaustion characterized by energy depletion. It resulted from prolonged and chronic exposure to stressors at the workplace which was not successfully managed [10]. Although a wide range of professions and ages are affected by burnout, demanding jobs such as healthcare professions appeared to have the lion’s share of this syndrome [11]. The current pandemic has increased globally the demand on healthcare services and HCWs encountered several stressors due to the nature of their work and their role in caring for COVID-19 patients [12]. These challenges included harmful working conditions, emotionally demanding patient contacts, witnessing COVID-19 related deaths, deep despondency, time pressure, extensive work hours, and work overload. All these factors are fueling HCWs’ burnout at an exponential rate [13]. The latter was more penetrating among those working in the frontlines of combating COVID-19 in hospitals [14]. Such an increased level of burnout might threaten the maintenance of a functioning healthcare workforce. In addition, several studies targeting burnout among HCWs have reported adverse consequences of this syndrome including the increased likelihood of individuals to develop psychiatric and physical illnesses. The psychological symptoms comprised depression, alcohol, and drug misuse, insomnia, appetite disturbances, suicidal ideas, while the physical symptoms could involve neck and back pain [15]. Of note, the impact of burnout is not limited to threatening the well-being of HCWs, but it can also affect other aspects such as the quality of health care delivered by HCWs, their professional efficacy and performance, as well as the patient safety. Lastly, the burnout can also trigger HCWs’ resignation and increase their reluctance to treat people as well as their early retirement. The latest may impede our recovery from the pandemic and can jeopardize the future global supply of the healthcare workforce [16]. In addition, such a syndrome can even increase the risk of medical errors [17]. A literature review on burnout and medical errors conducted by Brown et al., reported that physicians suffering from burnout experienced depression and substance dependency. It is worth mentioning that HCWs’ burnout may reach a tipping point with the emergence of new virulent mutants of the virus, the escalating death toll, low vaccination coverage, and slow vaccine rollout. Moreover, important burnout and stress levels are anticipated to persist long after the pandemic.

As for health systems, low-to-middle income countries such as Lebanon may have fewer buffering resources and capacity against shocks from the COVID-19 pandemic [18]. In addition, Lebanon, this small Middle-Eastern country, is crippled by several overlapping and multilayered crises with unique magnitude on the stability of its human capital [19]. Prior to the COVID-19 pandemic, a humanitarian crisis revealed by the influx of more than one million Syrian refugees to Lebanon has affected various sectors, mainly the economy, health, and education, and burdened the country’s health care system and the health care providers as well. Then, the abrupt stop in capitals inflows triggered a severe economic crisis which was the worst financial crisis recorded in the country’s history. According to the World Bank, this crisis ranked in the top ten, possibly top three, most severe crises episodes globally since the mid-nineteenth century [20, 21]. In addition, the country crumbles amidst an escalated political crisis and the COVID-19 pandemic that continues to impact many aspects of life with 181,503 cases and 1455 deaths as of December 31, 2020 [22].

In such conditions, the mental health of the general Lebanese population, particularly the one of HCWs has been undoubtedly severely affected. Several studies conducted in Lebanon have examined burnout syndrome among residents and nurses before the pandemic [23–25]. However, in the context of COVID-19, the psychological and mental health impacts of COVID-19 in Lebanon have predominantly been evaluated within specialties and single institutions [26]. Furthermore, no previous study in Lebanon has focused on assessing burnout among HCWs using national, large, diverse, and multi-institution samples. Therefore, it is of great interest to assess the prevalence of burnout among Lebanese HCWs at the national level during the COVID-19 pandemic. Such information is necessary to qualify future interventions that assist stakeholders to think about strategies to reduce stressors and take care of these HCWs, as well as to direct preventive measures or prophylaxis of these morbidities during and after the pandemic.

The present study aims to assess the prevalence of burnout among Lebanese HCWs in the context of COVID-19 and to identify its associated factors.

## Methods

### Study design and population

This quantitative cross-sectional study was conducted over a period of 2 months extending from the first of November to the end of December 2020 among Lebanese HCWs using a snowball sampling technique. Eligible participants included all Lebanese HCWs practicing in all operating healthcare facilities located in the eight Lebanese governorates (Bekaa, Baalbeck-Hermel, South, Nabatyeh, Akkar, North, Beirut, and Mount Lebanon). Participants were electronically invited to participate since the Lebanese government recommended minimalizing face-to-face interaction. All HCWs currently working in active hospitals and who had access to the internet were eligible to participate in this study. The term “HCW” was defined as any regulated health professional and any staff member, or other essential caregivers currently working in a health care facility. Therefore, we included doctors, nurses, paramedics, and administrative staff. This study excluded HCWs who are not practicing, those who were out of the country at the time of the survey, retired HCWs, those who suffered from and those who refused to participate.

### Sample size calculation

To calculate the required sample size for this study, the Rao soft digital sample size calculator was used. Supposing that there are around 50,000 registered HCWs and 40,000 of them are actively practicing at the health facilities level and considering a 95% confidence level and an estimation of absolute error of 5%, all previous information yielded to estimate a least required sample size of 381 participants. The required sample size was achieved at an early stage before the closure of response acceptance (January 1st, 2021). Of note, we achieved a large sample size (1751 participants) which was 4.59 times higher than the required one. Therefore, this could reduce sampling error and increase the study power.

### Ethical consideration

Participants were aware of the purpose of the study. Their participation was entirely voluntary, and they were free to withdraw at any time. Since the study has no foreseeable risks and the study design assured adequate protection of the participants’, written consent was obtained in an electronic format before enrolling. In addition, all information were gathered anonymously and handled confidentially. None of the survey questions asked for information that could harm the participant in any way and no reward was received by respondents in return for the participation. All methods were performed following the relevant guidelines and regulations after being reviewed and approved by the Ministry of Public Health (MOPH).

### Instrumentation

Data were collected using an Arabic, online, self-administered questionnaire, developed using a google form, which included closed-ended questions. The questionnaire consisted of four sections: (a) basic sociodemographic characteristics, (b) exposure to COVID-19 covariates, (c) occupational factors, and (d) the measurements.

The first section collected basic sociodemographic data of the participants, including gender, age, marital status, job category, urbanicity, health status, and living conditions. It also included questions about the history of medical illnesses and the health status of people living with the participant. Participants were also asked about the type and the location of the health facility where they worked.

The second section covered the topic of exposure to COVID-19. HCWs were queried to answer on whether they have (a) been tested for COVID-9, (b) ever been infected with COVID-19, (c) have a family member relative or colleague ever been infected by COVID-19. In addition, the status of infection protection was assessed with two items: “adherence to infection control procedures?” and “satisfaction with the hospitals’ infection control precautionary measures?”. Each of these variables was answered on a yes or no basis.

The third section comprises occupational factors such as the type of health facility where the HCW practice, working hours, being frontline HCW, and if the respondent treats or cares for COVID-19 patients. Surveyed HCW was also queried about his sleep patterns during the pandemic.

The fourth section included two scales:

1. The perceived threat and altruistic acceptance of the risk questionnaire.
  This instrument, which comprises 10 items, was developed by Chong et al. to assess the risk perception of COVID-19. Nine of its items assessed the risk perceived by HCWs towards the COVID-19 threat and one item evaluated the risk acceptance of caring for COVID-19 cases termed as “altruism” [27]. Rating of items was specified based on a five-point Likert scale (1 = strongly disagree, 2 = disagree, 3 = neutral, 4 = agree, 5 = strongly angry). Responses were dichotomized into positive responses ‘agree’ or ‘strongly agree’, while ‘strongly disagree’, ‘disagree’, and ‘not sure’ were considered negative. This scale showed good reliability in numerous studies and was previously used in the Lebanese context [28]. The Cronbach alpha of this scale in the current study was 0.721.
2. The Arabic version of Copenhagen Burnout scale A-CBI:
  The Arabic version of CBI, validated by Youssef et al. among HCWs [29], which consisted of 19 items, was used. It evaluates personal-related (6 items), work-related (7 items), and client-related (6 items) burnout. Ratings were given based on a five-point Likert scale. Each item was scored from 0 to 100 (0 = never, 25 = seldom, 0 = sometimes, 75 = often, 100 = always). Of note, some questions were answered using another five-point Likert scale (to a very high degree, to a high degree, somewhat, to a low degree, to a very low degree). However, the same scoring as for the first scale was adopted. Mean items score was calculated per scale. Each scale score depicts the direction indicated by its name. To avoid the stereotypy in HCWs’ responses, questions of CBI are mixed with other topics. To evaluate the prevalence of burnout among HCWs, a cut-off of 50 was used. A burnout level less than 50 indicated a low burnout level or its absence while a score higher than 50 indicated a moderate (50–75) and high (75–100) burnout level accordingly. In our study, the Cronbach’s alpha of this scale was equal to 0.879. Of note, a reverse coding was performed to item number 7 “Do you have enough energy for family and friends during leisure time” in the work-related burnout score.

### Data collection

After approval of the research by the Ministry of Public Health, focal persons working in health facilities were contacted via phone call and notified about the survey and its purpose. Upon their agreement to participate, an online questionnaire using a Google form was sent to them. They were also requested to disseminate the link of the study among their colleagues in the health care facility. An introductory note along with the questionnaire, which explained the intent of the survey, and an assurance that strict anonymity and confidentiality of data will be maintained. It also contains specific instructions for filling out the questionnaire. Though this has an optional field for the e-mail address of the respondents, we did not choose this option. Thus, the identity of the respondents was not known to any of the investigators. It took around 9 min to fill out this survey. Request to participate was sent twice at an interval of 10 days.

### Statistical analysis

The collected data were entered and analyzed using the statistical software SPSS (Statistical Package for Social Sciences), version 24.0. Descriptive statistics were reported using frequency with percentages for categorical variables and mean and standard deviation for continuous variables. Since missing data constituted < 10% of the total database, then it was not substituted. Before analysis, the distribution of each item of CBI and threat perception scale was checked for normality. Mean scores (mean ± SD) in personal, work-related, and client-related (pandemic related) domains were calculated using the 0- to 100-point scale. To assess the prevalence of burnout among Lebanese HCWs, CBI cut-off of 50 was used and CBI scores were categorized as follows: scores less than 50 are considered “no/low”, scores of 50–74 are considered ‘moderate’, 75–99 are high, and a score of 100 is considered severe burnout. A bivariate analysis was conducted using the Chi2 test to test the association between CBI and categorical variables. Spearmen correlation was used for linear correlation between continuous variables. All variables that showed a *p*-value < 0.2 in the bivariate analysis were included in the model as independent variables. Multinomial logistic analyses were performed. Statistical significance level was set at *p*-value < 0.05.

## Results

### Sociodemographic characteristics of the surveyed HCWs

A total of 1751 HCWs participated in this study. Table 1 displays the baseline characteristics of the surveyed HCWs. The majority were female (67.3%), married (62.6%), and aged between 30 and 49 years (49.4%). More than half of the participants were living in urban areas (61.1%) and working in private health facilities (64.9%), located mainly in Mount-Lebanon governorate (30.8%). The bulk of surveyed HCWs (78.5%) had a good health status and nearly half of them had dependent children or are living at home with an elderly or a family member suffering from comorbidities (Table 1).

**Table 1:** Baseline information of the surveyed Lebanese health care workers (*N* = 1751)

	*n*	%
Gender		
Male	572	32.70
Female	1179	67.30
Age (years)		
18–29 year	636	36.30
30–49 year	874	49.40
≥ 50 years	221	13.70
Marital status		
Single	606	34.60
Married/engaged	1096	62.60
Other (divorced or widowed)	49	2.80
Residence (urbanicity)		
Rural	681	38.90
Urban	1070	61.10
Occupation		
Physician	320	18.30
Nurse	908	51.90
Other*	523	29.8
Health facility type		
Public	615	35.10
Private	1136	64.90
Location of health facility		
North & Akkar	241	13.80
Mount Lebanon	540	30.80
Beirut	313	17.90
South & Nabatyeh	285	16.30
Bekaa & Baalbeck-Hermel	372	21.20
Health status		
Fair and Below	381	21.80
Good and above	1370	78.20
Presence of child at home		
No	779	44.50
Yes	972	55.50
Presence of elderly people at home		
No	923	52.70
Yes	828	47.30
Living with a family member with comorbidities		
No	782	44.70
Yes	969	55.30
Working in the frontline		
No	735	42.00
Yes	1016	58.00
Following up or caring of COVID-19 case		
No	843	48.10
Yes	908	51.90
Ever tested for COVID-19		
No	389	22.20
Yes	1362	77.80
Personal history of COVID-19 diagnosis		
No	1385	79.10
Yes	366	20.90
Family member ever diagnosed with COVID-19		
No	1059	60.50
Yes	692	39.50
Colleague/friend ever diagnosed with COVID-19		
No	134	7.70
Yes	1617	92.30

*n* frequency, % percentage, *other included all other health professions: pharmacists, midwives, laboratory technicians, etc.

### Descriptive of the scales used in the study

#### CBI items

As seen in Table 2, the items related to work burnout had the highest means such as feeling tired from every working hour (68.11 ± 15.53), being frustrated from work (67.96 ± 16.43), and feeling worn out at the end of the working day (67.804 ± 16.636). The items related to personal burnout came in the second rank, such as being emotionally or physically exhausted, feeling worn out, feeling tired or susceptible to illness, and thinking about the lack of ability to take anymore. Regarding internal consistency, the used scales showed good reliability and the α-values obtained of subscales ranged between 0.721 and 0.903, indicating good reliability: CBI (α = 0.861), PB (α = 0.814); WB (α = 0.903); CB (α = 0.834) and TP (α = 0.721). The overall CBI had a mean of 63.65 (SD = 21.32) while the value for the TP scale was 35.38 (SD = 2.66). The highest burnout mean was shown in WB aspect (67.53 ± 17.15) followed by PB (65.09 ± 17.33) and CB (63.65 ± 21.32). The normality of all used scales and subscales was assumed since skewness and kurtosis were lower than 1 and the sample size was larger than 300.

**Table 2:** Descriptive statistics of CBI and threat perception

	Mean	Std. deviation
Personal burnout (nb of items = 6, α = 0.814)	**65.09**	**17.23**
How often do you feel weak and susceptible to illness?	65.43	17.21
How often you are emotionally exhausted?	64.52	17.09
How often do you feel worn out?	65.67	17.52
How often do you feel tired?	63.89	17.15
How often you are physically exhausted?	64.13	17.03
How often do you think:” I can’t take it anymore”?	66.93	14.57
Work burnout (nb of items = 7, α = 0.903)	**67.53**	**17.15**
Is your work emotionally exhausting?	67.43	16.45
Does your work frustrate you?	67.96	16.43
Do you feel worn out at the end of the working day?	67.81	16.64
Do you feel that every working hour is tiring for you?	68.11	15.53
Are you exhausted in the morning at the thought of another day at work?	67.54	16.56
Do you feel burnt out because of your work?	67.11	15.33
Do you have enough energy for family and friends during leisure time?	66.76	16.11
Client Burnout (nb of items = 6, α = 0.834)	**57.70**	**22.10**
Do you find it frustrating to work with clients?	56.05	21.71
Does it drain your energy to work with clients?	56.44	21.49
Do you find it hard to work with clients?	55.25	22.39
Do you sometimes wonder how long you will be able to continue working with clients?	52.69	21.10
Do you feel that you give more than you get back when you work with clients?	55.57	22.13
Are you tired of working with clients?	70.09	21.01
Threat perception (nb of items = 10, α = 0.721)	**35.38**	**2.66**

α Cronbach alpha, *nb* number

#### Threat perception and altruistic acceptance of risk during the outbreak

**Fig. 1:**
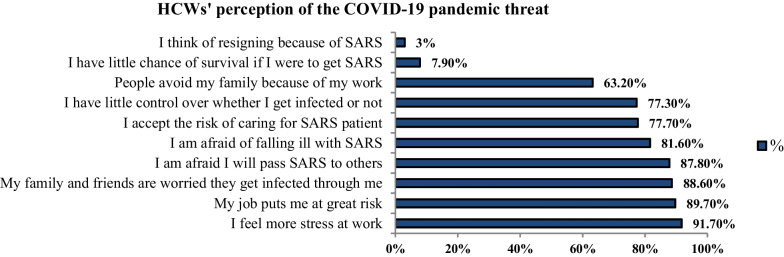
HCWs’ perception of the COVID-19 threats

Nearly 90% of surveyed HCWs believed that their job was putting them at risk, felt extra stress at work, and were afraid to transmit the COVID-19 to others. In addition, 81.6% were afraid of being infected by COVID-19 and 77.3% of them felt that they had little control over being infected or not. Only 7.9% of participants perceived a little chance of survival if they got infected and 3% thought about resigning because of COVID-19. Also, 88.6% of surveyed HCWs considered that their families and friends feared getting ill because of them. More than half of respondents were concerned about the avoidance of their families by others due to the nature of their work. As for altruistic acceptance of risks, most participants (77.7%) accepted taking the risk of caring for COVID-19 patients (Fig. 1).

### Prevalence of burnout among Lebanese HCWs

**Fig. 2:**
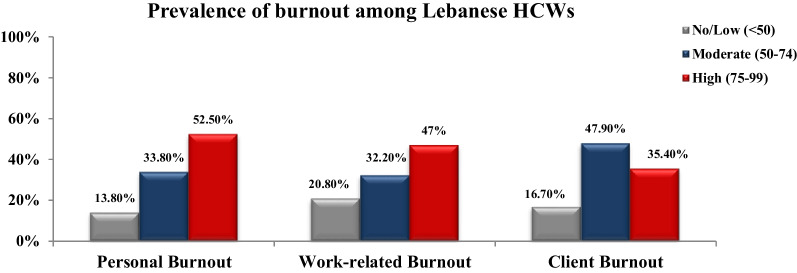
Prevalence of the three aspects of burnout among Lebanese HCWs

Out of all, personal burnout was detected in its moderate and high-level aspects among 86.3% of Lebanese HCWs with more than half (52.5%) of them exhibiting a high level of personal burnout (CBI score > 75). As for WB, a moderate and high levels of burnout hits around 79.2% of HCWs. Notably, 47% of them suffered from a high level of WB. Moderate and High levels of CB were prevalent among 83.3% of HCWs and 35.4% of them experienced a high level of CB. Of note, only a small percentage (≤ 20%) of surveyed HCWs, experienced a low level of burnout or did not experience burnout at all (Fig. 2).

### Factors associated with personal burnout among HCWs

1. Moderate PB vs no/low PB:
  Being married compared to single/divorced (aOR = 1.60; 95% CI 1.21–1.90), being a physician compared to other HCWs’ occupations (aOR = 2.59; 95% CI 2.01–6.72), having a dependent child(aOR = 1.15; 95% CI 1.61– 2.19), working in the frontlines (aOR = 1.82; 95% CI 1.62–5.12), being diagnosed with COVID—19 (aOR = 1.92; 95% CI 1.28–3.48) or having a colleague diagnosed with COVID-19 (aOR = 2.24; 95% CI 1.51–6.24), higher COVID-19 threat perception (aOR = 1.52; 95% CI 1.22–3.27), and limited sleeping hours (aOR = 1.85; 95% CI 1.12–3.92) were significantly positively associated with moderate personal burnout compared to no/low PB (Table 3, Model 1).
2. High PB vs no/low PB:
  As for sociodemographic factors, being female (aOR = 1.78; 95% CI 1.25–2.77), married compared to single/divorced (aOR = 1.51; 95% CI 1.22–3.18), having a poor health status (aOR = 1.80; 95% CI 1.11–3.55) were significantly positively associated with high personal burnout compared to no/low burnout. In addition, having a dependent child (aOR = 1.51; 95% CI 1.13–4.89) or an elderly at home (aOR = 2.09; 95% CI 1.28–5.56), having a family member suffering from comorbidities (aOR = 1.62; 95% CI 1.24–5.53), and a low income compared to high/moderate outcome (aOR = 3.43; 95% CI 1.38–4.62), were significantly positively associated with high personal burnout compared to no/low burnout. However, older age (> 30 years) compared to younger age (≤ 30 years) (aOR = 0.81; 95% CI 0.54–0.93) was negatively associated with high PB compared to no/low PB.
  As for exposure factors, working in the frontlines (aOR = 1.36; 95% CI 1.12–4.01), being diagnosed with COVID-19 (aOR = 2.17; 95% CI 1.82–3.66) or having a colleague diagnosed with COVID-19 (aOR = 1.66; 95% CI 1.47–5.93) and higher COVID-19 threat perception (aOR = 1.42; 95% CI 1.20–3.19) as well, increased the likelihood of high PB compared to no/low burnout. In terms of work-related factors, being a physician compared to other HCWs’ occupations (aOR = 2.83; 95% CI 1.12–6.98), working in a public health facility (aOR = 3.29; 95% CI 2.84–5.32), extensive working hours (aOR = 2.03; 95% CI 1.48–4.01), and limited sleeping hours (aOR = 1.85; 95% CI 1.12–3.92), were significantly positively associated with high personal burnout compared to no/low burnout. However, altruism (aOR = 0.46; 95% CI 0.33–0.71) was associated negatively with high PB (Table 3, Model 2).

**Table 3:** Multivariable analysis: multinomial regression for the personal burnout among HCWs

Variable	*p*-value	aOR	95% Confidence interval	
			Lower bound	Upper bound
Model 1: Personal burnout (moderate vs low/no burnout)				
Age (> 30 year vs < 30 year*)	0.220	1.820	0.715	2.767
Marital status (married vs single/divorced*)	**0.049**	**1.604**	**1.212**	**1.902**
Occupation (physicians vs other HCWs *)	**0.022**	**2.590**	**2.013**	**6.724**
Hospital type (private vs public*)	0.257	0.612	0.558	1.917
Health insurance (private vs public*)	0.336	.719	0.841	5.933
Gender (female vs male*)	0.213	1.018	0.886	1.386
Health condition (poor vs good*)	0.207	1.773	0.946	2.290
Presence of child at home (yes vs no*)	**0.026**	**1.154**	**1.606**	**2.194**
Presence of elderly at home (yes vs no*)	0.181	0.987	0.831	1.978
Family member with comorbidities	0.231	0.866	0.710	2.012
Working in the frontline (yes vs no*)	**0.025**	**1.822**	**1.615**	**5.117**
Tested for COVID-19 (PCR test) (yes vs no*)	0.601	0.957	0.713	6.816
Diagnosed as COVID-19 case (yes vs no*)	**0.016**	**1.915**	**1.277**	**3.481**
Family member with comorbidities (yes vs no*)	0.183	0.897	0.211	3.822
Colleague diagnosed with COVID-19 (yes vs no*)	** < 0.001**	**2.238**	**1.509**	**6.239**
Threat perception scale	**0.038**	**1.517**	**1.218**	**3.271**
Altruistic (yes vs no *)	0.149	0.435	0.377	1.615
Extensive working hours (yes vs no*)	0.198	0.886	0.765	1.245
Income (low vs high*)	**0.031**	0.698	0.437	0.721
Sleeping hours (less than 6 h vs > 6 h *)	**0.011**	**1.854**	**1.123**	**3.922**
Model 2: Personal burnout (high vs low/no burnout)				
Age (< 30 year vs > 30 year vs *)	**0.011**	**1.812**	**1.541**	**2.929**
Marital status (married vs single/divorced*)	**0.041**	**1.513**	**1.218**	**3.183**
Occupation (physicians vs other HCWs *)	**0.003**	**2.832**	**1.122**	**6.983**
Hospital type (public vs private *)	**0.038**	**3.291**	**2.837**	**5.316**
Health insurance (private vs public*)	0.195	1.713	0.896	2.357
Gender (female vs male*)	**0.025**	**1.776**	**1.250**	**2.075**
Health condition (poor vs good*)	**0.029**	**1.804**	**1.114**	**3.549**
Presence of child at home (yes vs no*)	**0.016**	**1.507**	**1.130**	**4.890**
Presence of elderly at home (yes vs no*)	**0.049**	**2.091**	**1.277**	**5.556**
Family member with comorbidities	0.327	1.017	0.715	1.445
Working in the frontline (yes vs no*)	**0.040**	**1.365**	**1.119**	**4.013**
Tested for COVID-19 (PCR test) (yes vs no*)	0.656	0.990	0.687	1.426
Diagnosed as COVID-19 case (yes vs no*)	**0.039**	**1.833**	**1.552**	**6.260**
Family member with comorbidities (yes vs no*)	**0.034**	**1.618**	**1.236**	**5.525**
Colleague diagnosed with COVID-19 (yes vs no*)	**0.015**	**1.664**	**1.476**	**3.925**
Threat perception scale	**0.026**	**1.420**	**1.196**	**3.189**
Altruistic (yes vs no*)	**0.011**	**0.457**	**0.328**	**0.712**
Extensive working hours (yes vs no*)	**< 0.001**	**2.031**	**1.476**	**4.012**
Income (low vs high*)	**0.041**	**3.435**	**1.377**	**4.615**
Sleeping hours (less than 6 h vs > 6 h *)	**0.023**	**2.513**	**1.428**	**5.811**

*Reference group, numbers in bold indicate significant *p*-value, aOR: adjusted odds ratio, Goodness of fit Pearson value = 2997.382, *p* < 0.001, Pseudo *R*^2^ = 13.9

### Factors associated with work-related burnout among HCWs

1. Moderate WB vs no/low WB:
  HCWs who were physicians compared to those with other healthcare occupations (aOR = 1. 95; 95% CI 1.34–4.41), those who worked in a public health facility compared to HCWs practicing private health facility (aOR = 1.82; 95% CI 1.42–3.27) and those who have a colleague diagnosed with COVID-19 (aOR = 2.31; 95% CI 1.97–3.44) were more likely to suffer from a moderate level of WB than from a no/low PB level (Table 4, Model 1).
2. High WB vs no/low WB:
  As for sociodemographic factors, being female HCW (aOR = 2.78; 95% CI 1.15–4.08), married (aOR = 2.20; 95% CI 1.40–3.98), aged 30 years old or less (aOR = 2.78; 95% CI 1.15–4.08) were significantly positively associated with high WB compared to no/low burnout. In addition, low income compared to high/moderate outcome (aOR = 3.01; 95% CI 1.52–7.31)), were significantly positively associated with high personal burnout compared to no/low burnout. However, older age (> 30 years) compared to younger age (≤ 30 years) (aOR = 0.81; 95% CI 0.54–0.93) was negatively associated with high PB compared to no/low PB.
  As for exposure factors, working in the frontlines (aOR = 2.17; 95% CI 1.82–5.66), having a colleague diagnosed with COVID-19 (aOR = 1.66; 95% CI 1.17–3.92) and higher COVID-19 threat perception (aOR = 2.42; 95% CI 1.20–5.19) as well, increased the likelihood of high WB compared to no/low burnout. In terms of work-related factors, being a physician compared to other HCWs’ occupations (aOR = 2.51; 95% CI 1.15–3.08), working in a public health facility (aOR = 3.19; 95% CI 2.11–6.32), extensive working hours (aOR = 2.71; 95% CI 1.85–4.79), and limited sleeping hours (aOR = 2.51; 95% CI 1.43–4.81), were significantly positively associated with high WB compared to no/low burnout. However, altruism (aOR = 0.65; 95% CI 0.41–0.61) was associated negatively with high WB (Table 4, Model 2).

**Table 4:** Multivariable analysis: multinomial regression for the work-related burnout among HCWs

Variable	*p*-value	aOR	95% Confidence interval	
			Lower bound	Upper bound
Model 1: Work-related burnout (moderate vs low/no burnout)				
Age (> 30 year vs < 30 year*)	0.210	1.530	0.902	2.183
Marital status (married vs single/divorced*)	0.248	1.442	0.715	1.987
Occupation (physicians vs other HCWs *)	**0.022**	**1. 950**	**1.338**	**4.405**
Hospital type (public vs private *)	**0.017**	**1.820**	**1.418**	**3.270**
Health insurance (private vs public*)	0.136	0.826	0.842	3.922
Gender (female vs male*)	0.318	1.183	0.886	3.663
Health condition (poor vs good*)	0.119	1.773	0.946	2.290
Presence of child at home (yes vs no*)	0.126	1.154	0.606	2.196
Presence of elderly at home (yes vs no*)	0.141	1.185	0.616	2.277
Family member with comorbidities	0.183	0.960	0.504	1.828
Working in the frontline (yes vs no*)	0.225	1.909	0.651	2.571
Tested for COVID-19 (PCR test) (yes vs no*)	0.668	0.985	0.534	1.816
Diagnosed as COVID-19 case (yes vs no*)	0.121	0.751	0.421	4.846
Family member with comorbidities (yes vs no*)	0.283	0.897	0.211	3.822
Colleague diagnosed with COVID-19 (yes vs no*)	**< 0.001**	**2.310**	**1.967**	**3.439**
Threat perception scale	0.208	1.657	0.941	4.712
Altruistic (yes vs no*)	0.301	0.435	0.377	1.615
Extensive working hours (yes vs no*)	0.091	0.681	0.465	3.451
Income (high vs low *)	0.131	0.698	0.437	2.712
Sleeping hours (> 6 h vs less than 6 h*)	0.171	0.654	0.713	2.917
Model 2: Work-related burnout (high vs no/low burnout)				
Age (> 30 year vs ≤ 30 year*)	**0.012**	0.770	0.608	0.974
Marital status (married vs single/divorced*)	**0.006**	2.203	1.401	3.983
Occupation (physicians vs other HCWs *)	**0.009**	2.513	1.147	4.983
Hospital type (private vs public*)	**< 0.001**	3.191	2.113	6.316
Health insurance (private vs public*)	0.371	0.885	0.676	1.157
Gender (female vs male*)	**0.025**	2.776	1.150	4.075
Health condition (poor vs good*)	0.222	1.044	0.704	1.549
Presence of child at home (yes vs no*)	0.306	1.507	0.813	3.890
Presence of elderly at home (yes vs no*)	0.131	3.099	0.977	6.556
Family member with comorbidities	0.927	1.017	0.715	1.445
Working in the frontline (yes vs no*)	**0.040**	**2.165**	**1.817**	**5.660**
Tested for COVID-19 (PCR test) (yes vs no*)	0.656	0.990	0.687	1.426
Diagnosed as COVID-19 case (no vs yes*)	0.069	0.833	0.552	1.260
Family member with comorbidities (yes vs no*)	0.324	1.618	0.836	3.525
Colleague diagnosed with COVID-19 (yes vs no*)	**0.015**	**1.664**	**1.176**	**3.924**
Threat perception scale	**0.026**	**2.311**	**1.816**	**5.189**
Altruistic (yes vs no*)	**0.011**	**0.651**	**0.408**	**0.612**
Extensive working hours (yes vs no*)	**< 0.001**	**2.713**	**1.847**	**4.792**
Income (low vs high*)	**0.011**	**3.014**	**1.522**	**7.314**
Sleeping hours (less than 6 h vs > 6 h *)	**0.023**	**2.513**	**1.428**	**6.811**

*Reference group, numbers in bold indicate significant *p*-value, aOR: adjusted odds ratio, Goodness of fit Pearson value = 2103.19, *p* < 0.001, Pseudo *R*^2^ = 11.4

### Factors associated with client-related burnout among HCWs

1. Moderate CB vs no/low CB:
  Working in the frontlines (aOR = 1.91; 95% CI 1.65–2.57), having a colleague diagnosed with COVID-19 (aOR = 2.31; 95% CI 1.97–3.24), and higher COVID-19 threat perception (aOR = 1.35; 95% CI 1.30–3.12), and limited sleeping hours (aOR = 1.85; 95% CI 1.12–3.92) were significantly positively associated with moderate personal burnout compared to no/low CB (Table 5, Model 1).
2. High CB vs no/low CB:
  Older age of HCW (> 30 years) (aOR = 0.77; 95% CI 0.61–0.97) and altruism (aOR = 0.52; 95% CI 0.41–0.87) were negatively associated with high CB compared to no/low burnout. However, working in the frontline (aOR = 2.01; 95% CI 1.50–4.13), threat perception (aOR = 1.42; 95% CI 1.50–4.13), extensive working hours (aOR = 2.13; 95% CI 1.49–4.00), less than 6 h sleeping hours (aOR = 1.57; 95% CI 1.21–3.11), and low income compared to high/moderate outcome (aOR = 1.91; 95% CI 1.42–3.27), were significantly positively associated with high client burnout compared to no/low burnout (Table 5, Model 2).

**Table 5:** Multivariable analysis: multinomial regression for the client-related burnout among HCWs

Variable	*p*-value	aOR	95% Confidence interval	
			Lower bound	Upper bound
Model 1: client-related burnout (Moderate vs low/no burnout)				
Age (> 30 year vs < 30 year*)	0.313	1.713	0.922	3.018
Marital status (Married vs single/divorced*)	0.418	1.314	0.507	1.807
Occupation (physicians vs other HCWs *)	0.152	2.590	0.839	2.705
Hospital type (private vs public*)	0.302	0.812	0.701	1.327
Gender (female vs male*)	0.308	1.183	0.886	4.643
Health condition (poor vs good*)	0.119	1.773	0.946	3.139
Presence of child at home (yes vs no*)	0.298	1.154	0.816	2.196
Presence of elderly at home (yes vs no*)	0.111	1.185	0.616	2.277
Family member with comorbidities	0.181	0.960	0.504	1.828
Working in the frontline (yes vs no*)	**0.025**	1.909	1.651	2.571
Diagnosed as COVID-19 case (yes vs no*)	0.112	2.351	0.814	4.846
Family member with comorbidities (yes vs no*)	0.283	0.897	0.211	3.822
Colleague diagnosed with COVID-19 (yes vs no*)	**< 0.001**	2.310	1.967	3.239
Threat perception scale	**0.048**	1.345	1.298	3.124
Altruistic (yes vs no*)	0.241	0.435	0.377	1.615
Extensive working hours (yes vs no*)	0.098	0.886	0.765	1.245
Income (Low vs high*)	0.126	0.898	0.753	1.942
Sleeping hours (> 6 h vs less than 6 h*)	0.271	0.854	0.723	1.914
Model 2: client-related burnout (High vs no/low burnout)				
Age (> 30 year vs < 30 year*)	**0.029**	**0.770**	**0.608**	**0.974**
Marital status (married vs single/divorced*)	0.152	1.513	0.772	4.003
Occupation (physicians vs other HCWs *)	0.112	1.093	0.981	2.832
Hospital type (private vs public*)	0.331	2.291	0.811	4.022
Gender (female vs male*)	0.205	1.776	1.115	2.075
Health condition (poor vs good*)	0.329	1.044	0.704	1.905
Presence of child at home (yes vs no*)	0.116	1.507	0.893	3.890
Presence of elderly at home (yes vs no*)	0.449	2.099	1.277	2.556
Family member with comorbidities	0.527	1.017	0.895	10.215
Working in the frontline (yes vs no*)	**0.021**	**2.013**	**1.502**	**4.131**
Diagnosed as COVID-19 case (yes vs no*)	0.308	1.833	0.552	3.260
Family member with comorbidities (yes vs no*)	0.141	1.618	0.936	2.145
Colleague diagnosed with COVID-19 (yes vs no*)	0.315	0.664	0.541	2.912
Threat perception scale	**0.031**	**1.420**	**1.196**	**2.189**
Altruistic (yes vs no*)	**< 0.001**	**0.517**	**0.412**	**0.866**
Extensive working hours (yes vs no*)	**< 0.001**	**2.131**	**1.476**	**4.002**
Income (low vs high*)	**0.006**	**1.905**	**1.415**	**3.271**
Sleeping hours (less than 6 h vs > 6 h vs *)	**0.023**	**1.573**	**1. 209**	**3.108**

*Reference group, numbers in bold indicate significant *p*-value, aOR: adjusted odds ratio

## Discussion

To fulfill their dedication to the medical profession's responsibilities and obligations, HCWs have done their utmost around the clock to save, diagnose, and treat COVID-19 patients. The outbreak of COVID-19 triggered widespread alarm about the potential of this unprecedented global crisis to increase the level of burnout among HCWs. Therefore, this study is the first national, large, diverse, and multi-institution study assessing the prevalence of burnout among HCWs and identifying its associated factors during the COVID-19 pandemic, using a validated scale. We believe that this study outlines interesting avenues for future research.

Our study found that around 80% of HCWs suffered from a moderate and high level of burnout comprising its three dimensions (personal, occupational, and patient-related). HCWs who were females, married, physicians, those who have a poor health status, and those who had specific living conditions (dependent child, elderly at home, family member with comorbidities, and a low income) were more likely to exhibit a high level of PB compared to no/low burnout. Additionally, frontline HCWs, those who were diagnosed with COVID-19 or had a COVID-19, and those who had a high perception of COVID-19 threat were more prone to suffer a high-level PB compared to no/low burnout as well. Working in a public health facility, extensive working hours, and limited sleeping hours were significantly positively associated with high PB. However, older age and altruism were negatively associated with high PB compared to no/low PB. The same factors were found either positively or negatively associated with a high level of WB compared to no/low WB except health status and living conditions (dependent child or family member). As for high CB level, older age of HCW (> 30 years) and altruism were negatively associated with high CB compared to no/low burnout. However, working in the frontline, threat perception, extensive working hours, sleeping hours (less than 6 h), and low income were significantly positively associated with high CB compared to no/low burnout. We found a high prevalence of burnout during the current pandemic compared to the pre-COVID-19 era. This is difficult to compare with previous literature as most studies had used different scales.

The burnout level found among Lebanese HCWs was alarming since more than three-quarters of them suffered from all aspects of burnout including personal (86.3%), work-related (79.2%), and client-related burnout (83.3%) in their moderate and high levels. The present study lacked a control group, but our results supported the findings of various studies regarding the potential of the pandemic to increase the level of burnout among HCWs. Similarly, a study conducted in China showed also that HCWs battling COVID-19 exhibited a high level of burnout [30]. However, the worrying prevalence of burnout found in our study was higher than the figures reported in previous studies conducted among HCWs in Asia during the COVID-19 pandemic as these studies disclosed that the prevalence of burnout in HCWs varies from 31.4% to 75% [30–33]. On the other hand, our results were in line with the findings of a study conducted among HCWs in Saudi Arabia, an Arabic country that owns one of the superlative healthcare systems in the Middle East, where 75% of Saudi HCWs suffered from burnout during the COVID-19 pandemic [33, 34]. In comparison with Europe which was severely impacted by COVID-19, several studies conducted by Barello et al. [35] and Lasalvia et al. [36] showed that Italian HCWs revealed significant work-related burnout symptoms and 56% of them reported emotional exhaustion [37]. In Portugal, according Duarte I et al. concluded that more than half of HCWs had symptoms of personal burnout [38]. Of note, owing to both the COVID-19 pandemic and severe economic crisis, such a high prevalence of burnout among Lebanese HCWs was anticipated. A similar finding was reported in the Libyan context, where 67.1% of HCWs suffered from emotional exhaustion due to the overlapping crises revealed by the pandemic and the civil war) [39]. It is noteworthy that the large difference across these studies in terms of the prevalence of burnout was expected since it could be resulting from the assessment methods’ heterogeneity, the disparity in burnout definitions, and regional differences [40].

A peculiar finding in our study was the drastic change in the dynamic of burnout during the pandemic. A huge increase in the prevalence of client-related burnout (pandemic-related) increased (83.3%) among HCWs was recorded in the era of COVID-19 compared to previous studies. For example, a pre-COVID-19 study conducted by Žutautienė et al. found a low prevalence of CB (35.1%) compared to the PB (44.8%) and WB (46.7%) burnout [41]. For the personal aspect of burnout, it was predominant. Of note, such a high prevalence of PB should not be linked completely to COVID-19 related factors given that other veiled risk factors could be associated with the increase of PB such as the economic factors.

In terms of factors associated with high PB, HCWs who were females, married, physicians, those who have a poor health status, and those who had specific living conditions (dependent child, elderly at home, family member with comorbidities, and a low income) were more likely to exhibit a high level of PB compared to no/low burnout. As for gender, while some studies agree on the fact that there is no real effect of this variable in the occurrence of burnout [42, 43], the data from the Medscape National Physician Report indicate that women physicians reported more often symptoms of burnout [41]. Our findings were also consistent with the results of an Italian survey that found higher levels of burnout in females and in young (aged < 30 years) HCWs [44]. Furthermore, a recent systematic review conducted by Prasad showed higher stress scores in US health organizations among women [45]. This could be explained by the high exposure to risk for female HCWs given their predominance in patient-facing roles, gender expectations in care, with high workloads at their home. As for occupation, despite that Burnout has been shown to occur in all kinds of jobs, our study showed that physicians were more likely to experience a high level of personal and occupational burnout than other HCWs. A similar finding was reported by Shanafelt et al. who reported that the incidence of burnout was 37.9% in physicians compared to 27.8% in the control population (*p* < 0.001) [46].

Another important aspect of personal and occupational burnout found in our study was the “married” marital status of HCWs. In addition, living conditions such as having a dependent child, elderly, or family member suffering from comorbidities at home were associated with a higher level of PB. The high prevalence of burnout could be resulting from family demands that denote possible familial-related responsibilities such as caring for family members who are sick, childcare, providing support, and managing complex familial relationships [47, 48]. Of note, family demands are commonly gendered as a result of imposed roles on women and there are discrepancies in who bears the brunt of such demands [49, 50]. Studies show that HCWs are tormented when it comes to balancing providing care for their patients and their families, which can lead to impairments in both spheres as well as to the burnout of HCWs [51].

Our study found that HCWs who worked in the frontline and those who were directly involved in the diagnosis and treatment of COVID-19 cases were more likely to express a high level of personal and occupational burnout. This was also reported in a study conducted in China comparing the mental health disturbances in physicians and nurses working at the frontline and the second-line healthcare workers [52]. In addition to burnout, other studies also disclosed a higher incidence and more severe symptoms of depression, anxiety, insomnia, and mental distress among frontline HCWs. Additionally, HCWs who had a high perception of COVID-19 threat were more prone to suffer from a high-level PB compared to no/low burnout as well. In this context, several studies focusing on conventional risks supported the potential association between perceived risk and negative outcomes for the individual, such as job burnout and low job satisfaction [53–55]. Overall, based on the results of previous research, we assumed that the perceived risk of being infected by COVID-19 at work and the fear of being infected were positively associated with a high level of burnout in its different aspects (personal, work-related, or patient-related). As for the history of COVID-19 infection and having a colleague infected with COVID-19, one multicenter cross-sectional survey also has shown that the history of contact with the patient was a risk factor doubling the risk of negative mental health outcomes such as anxiety and depression during the COVID-19 pandemic [56].

Another peculiar finding in our study was that HCWs practicing in a public health facility were more likely to express a higher level of PB and WB. This could be explained that limited work resources may increase job demands and the negative psychological/physiological costs associated with them, such as burnout which is the case of public health facilities [57]. However, such a finding was anticipated since public hospitals were the first facilities to be mobilized by the ministry of public health for the fight against COVID-19.

Age and altruism emerged in our study as protective factors where burnout levels in HCWs tend to decrease with increasing age and with altruism. This could be due to the more “perfectionistic” and empathic approach in younger HCWs [58]. As for altruism and its negative association with burnout, previous theories in psychology considered that altruistic behavior has dominance over negative emotions and anxiety. This will increase engagement in altruistic activities in challenging situations [59].

Extensive working hours and insufficient sleeping hours were also found associated with high level of burnout in all its aspects. This could be understood since the workload imposed by the pandemic leads to long working hours and short sleeping hours which are common factors associated with burnout. A study assessing the relationship between sleeping hours and burnout showed that the odds ratio of work‐related burnout doubled when hours exceeded 60 h, tripled when hours exceeded 74 h, and quadrupled when hours exceeded 84 h [60].

Lastly, low income was found as a factor increasing burnout in all its aspects. As known, socioeconomic status can refer to a person's quality of life as well as the opportunities and privileges they have in society. Several studies reported that financial strain was associated with high burnout [61] and socioeconomic status was found as a constant and reliable predictor of a wide range of life outcomes, including physical and mental health. However, some issues should be highlighted in the case of Lebanese HCWs who faced an unprecedented economic crisis. The latter has affected severely HCW’s income because of the depletion of the Lebanese currency. This has increased HCWs’ feelings of insecurity towards their work which could occupy their mind and make them lose focus. In addition, they can feel more hopeless and dissatisfied with their work and their patients. It is worth mentioning that having a low income in our study referred to the current self-perceived economic situation. The finding that HCWs who experienced a deteriorated economic situation (low income) during the study time also reported increasing burnout levels indicates that subjective economic difficulties might have an impact on burnout.

Although the relationship between burnout and the COVID-19 pandemic has been unveiled in this study, several potential factors associated with burnout among Lebanese HCWs were not investigated in our study. Hence, further research about other risk factors that could be incremental, factors related to economic factors, and how to alleviate burnout symptoms among HCWs fighting against COVID-19 is still needed.

### Limitations

There are several limitations in this study that should be addressed. First, the cross-sectional design of the study limits our ability to infer causal relationships. In addition, the collected data were also based on self-reported information which makes it prone to social desirability and might lead to underestimating some associations. Secondly, selection bias is possible due to the sampling technique used for data collection which limits the generalizability of the findings. Thirdly, our study data were collected using an online questionnaire. Although a substantial number of HCWs from different regions across Lebanon during the current outbreak of COVID-19 were able to participate and the good quality of data collected by online surveys, some drawbacks related to the online nature of the survey should be acknowledged. HCWs with who were busy with higher levels of workload, and possibly with a higher risk of being infected at work, may not have the time to fill out the survey. Additionally, HCWs with limited internet access may not have taken part in this study. Fourthly, we were unable to assess the pandemic's impact on burnout due to a lack of data on pre-COVID burnout among Lebanese HCWs using the same assessment tool. Lastly, further studies following up on the burnout of Lebanese physicians would be recommended in the future to confirm our results, especially since several waves of COVID-19 have been recorded since December 2020.

### Implications for clinical practice and research

The present study has relevant practical implications, in terms of burnout prevention, for hospitals and HCWs. Occupational risks were found in several studies to be associated with a higher workload. This could be explained that the threatening situations requiring additional efforts and tasks to be managed [62]. Hence to prevent burnout among HCWs in the hospital setting, we should consider the perceived threat of being infected by COVID-19 as an additional work demand for HCWs requiring an investment of further energies at physical and psychological levels. Hence, hospitals were encouraged to adjust the balance between job demands and available resources. This includes ensuring the availability of adequate protective equipment and effective safety-related policies, exchange of reliable information about COVID-19 risk across HCWs in addition to monitoring of implemented precautionary measures. On the one hand, the latter will allow controlling the work environment and effectively achieving safety at the workplace, thus allowing HCWs to feel safe and able to cope efficiently with the perceived threat of COVID-19. This will reduce pandemic-related burnout among them. On other hand, the alarming level of burnout unveiled among Lebanese HCWs represented only the tip of the iceberg where migration of a huge number of HCWs especially physicians and nurses were noticed in the previous months. Projections show that the health workforce shortage is expected to increase over the coming months. The latter could threaten the patient’s quality of care and the overall healthcare system. Such a high level of burnout underlines the urgent need that government and health facilities address this comorbidity through enacting proactive policies, providing critical leadership and funding for burnout prevention programs. A collaborative effort between national and institutional leadership will improve burnout management during this pandemic and better prepare us for the future. Since the health care workforce is an indispensable part of the economic growth and resilience of a nation, policymakers should be pragmatic in supporting funding for burnout prevention programs. Lastly, the long-term effects of the current pandemic need to be assessed later.

## Conclusion

The alarming level of burnout detected among Lebanese HCWs in all its three aspects (personal, occupational, and client-related) calls for urgent action. Health authorities should be proactive and address the factors associated with burnout unveiled in our study. Enacting and implementing preventive policies and effective evidence-based interventions are highly required to cultivate wellness among HCWs to reduce their burnout. This could slow down the ongoing attrition of HCWs, prevent possible detrimental consequences for HCW’s well-being and ensure all patients receive quality care from motivated and hopeful healthcare providers. Forthcoming studies that investigate additional situational and individual factors that may affect burnout are recommended.

## Data Availability

The datasets generated during the current study are not publicly available but are available from the corresponding author on reasonable request with the permission of the Ministry of Public Health, the clinical trial program.

## Abbreviations

COVID-19: Coronavirus disease 2019
CBI: Copenhagen Burnout Inventory
TP: Threat perception scale
HCW: Health care worker
PB: Personal burnout
WB: Work-related burnout
CB: Client-related burnout
MOPH: Ministry of Public Health
aOR: Adjusted odds ratio
EMR: Eastern Mediterranean Region
